# Constructing an experiential education model in undergraduate radiology education by the utilization of the picture archiving and communication system (PACS)

**DOI:** 10.1186/s12909-019-1827-0

**Published:** 2019-10-21

**Authors:** Yingqian Chen, Keguo Zheng, Shanshan Ye, Jifei Wang, Ling Xu, Ziping Li, Quanfei Meng, Jianyong Yang, Shi-Ting Feng

**Affiliations:** 1 Department of Radiology, The First Affiliated Hospital, Sun Yat-sen University, 58th, The Second Zhongshan Road, Guanzhou, 510080 China; 2 Faculty of Medicine and Dentistry, University of Western Australia, Perth, Australia

**Keywords:** Radiology education, Undergraduate students, Experiential education, PACS

## Abstract

**Background:**

Medical education in China is in a transitional period, from passive learning models to experiential education. We modified an experiential education method for radiology education. The aim of this study is to evaluate the effect of this method on undergraduate radiology education.

**Method:**

With the help of the picture archiving and communication system (PACS) and RadiAnt DICOM Viewer, we modified an experiential education method that simulates similar working conditions for undergraduate medical students to formulate radiology diagnosis similar to clinical radiologists. A total of 101 students were allocated into either the experiential education group or the control group. The final examination scores and a 5-point Likert scale self-assessment questionnaire of radiologic skills were collected from all the students as an objective assessment and a subjective assessment respectively. A questionnaire was also used to assess the satisfaction with the experiential model in the experiential education group. Mann-Whitney U test was used to compare the ranked data, and *t*-tests were used to compare the numeric data.

**Results:**

The experiential education group demonstrated significantly higher scores (7.4 ± 1.3) compared to the control group (6.7 ± 1.5, *p* < 0.05) in the question type “description and diagnosis”. The self-assessment questionnaire indicated that the experiential education was related to increased familiarity with the diagnosis thinking principle and the sequences and reconstruction methods of computer tomography (CT) imaging, which also strengthen participants’ self-confidence to perform future clinical work (*p* < 0.05). The self-assessment questionnaire in the experiential education group showed that the majority of students were satisfied with the organization (82.5%), interactivity (85%) and quality (85%) of the learning activity. Most students found this model of learning to be helpful for studying radiology (85%) and for understanding anatomy (90%).

**Conclusion:**

Compared with the traditional radiology education approach, the experiential education method showed greater efficacy in improving students’ analysis and diagnostic skills and their self-confidence.

## Background

Radiology is one of the key components in basic medical education, which serves as a bridge between anatomy and the clinic. Like the other areas of medical education, radiology education is facing a challenge of transitioning from passive learning to both interactive teaching and experiential learning [1, 2].

As the field of radiology expanded, radiology education experienced a revolution. Doctors used to carry plain films and teach using projectors or view boxes as plain films were the main diagnostic method in Radiology during the 1970s. Since the introduction of computed tomography (CT) and magnetic resonance (MR) imaging in the late 1980s, the increase in image data volume associated with these imaging modalities led to greater demands for compatible data storage platforms. Thus, the picture archiving and communication system (PACS), which can store, retrieve, distribute, analyse and digitally process medical images, has become an indispensable tool in today’s clinical work [3–5]. However, limited by the hardware and software conditions, the use of PACS in radiology training has remained somewhat limited [6, 7].

Currently, most radiology education continues to rely heavily on textbooks and traditional computer media such as PowerPoint or Word documents, both of which are lacking in student interactions [8]. There is little chance for a medical student to read the whole images like a real radiologist in class. It is often a challenge for them to grasp the concept of 3-dimensional (3D) gross anatomy, as well as a holistic view of diseases [9]. As a result, some students may struggle to independently identify abnormal findings and to analyse and formulate radiologic diagnoses. Previously, only limited final-year medical students demonstrated satisfactory basic radiology interpretive skills, which urged us to look for a more effective method [10].

A variety of radiology education methods have been previously reported, including problem-based learning, case-based learning, and team-based learning [11–13]. Unlike these previously studied conventional methods, under the concept of learning from experience, we modified a new experiential education method that enables students to practice radiology interpretation and diagnosis by taking on the radiologist’s role in a simulated environment. The whole typical cases instead of specific layers are shown to the student by using the PACS. Students are allowed to read the whole images as well as doing some basic reconstruction freely and find out the specific image characteristics by themselves. During this process, students can access the PACS and the clinical information, integrating both clinical knowledge and 3D reconstruction ability, which is essential for formulating radiological diagnoses. It is our first time to introduce the concept of experiential education into radiology education. We try to evaluate the effect of this method on undergraduate radiology education.

## Methods

### Image acquisition

Raw CT and MR data were copied directly from each machine or the PACS. Data were stored in DICOM (Digital Imaging and Communication in Medicine) format, which is a standard international multi-vendor format. To protect patients’ privacy, patient information was de-identified where their name and medical record numbers was removed. Digital images were then transferred into a teaching file.

### Hard- and software

Each student had a personal computer connected to the web server to download a case. The RadiAnt DICOM Viewer (version 4.0.3) was used as the teaching software. The software is user friendly, which enabled students to read images freely on their own computers.

### Subjects

All fourth year medical students with clinical medicine major (eight-year program) from the Medical School of Sun Yat-sen University were included in this study. One of three classes was randomly chosen as the experiential class and received experiential education. The control group consisted of students in the remaining two classes.

### Experiential education model

Following theoretical courses for a specific system, all students in the experiential education group and control group underwent a practical course of similar contact hours. The ratio of theoretical and practical courses is 3:2. An average of 4–5 cases for each system, altogether 60 cases were presented to students in the experiential education group, along with the corresponding medical history, physical examination results and laboratory test results. These cases are all the typical cases of each system. For example, typical CT images of lobar pneumonia and hematogenous pulmonary tuberculosis would be chosen when teaching the respiratory system. Students were allowed to read images freely and provided image descriptions and diagnoses within approximately one hour. The software enabled students to do basic operations with the images, such as adjusting the window width and level, comparing different sequences, and performing multiple planar reconstruction (MPR) or 3D reconstruction, just as what the radiologist could do in the medical PACS system. Students have received essential training for the software to ensure the exercise. Students then shared their findings and diagnoses in open discussions. It was then the role of the lecturer and the 3 teaching assistants to guide students in making complete and detailed image descriptions and correct diagnosis. In the last session of the class, the lecturer would summarize the reading points of the cases and the knowledge points of this system.

Meanwhile, the students in the control group still received the teaching by reading the typical imaging layers with the traditional computer media including PowerPoint and Word documents. The basic skills of reading images like the the reconstruction method and choosing the proper window width and level are taught only in theory. The students in the control group also had the opportunity to receive the guidance by the teacher if they had any questions.

### Assessment

After one semester of class, final examination was taken, which combining 30 multi-choice questions and 10 “image description and diagnosis” short answer type questions. The scores were collected as objective assessments. To provide a subjective assessment of radiologic skills, all of the students were invited to complete a self-assessment radiologic skills questionnaire (Additional file 1). The students in the experiential education group were also invited to complete a questionnaire assessing their satisfaction with the experiential learning condition (Additional file 2). Both of the self-made questionnaires used a 5-point Likert scale. The questionnaire used in the study was developed for this study and has not previously been published elsewhere.

### Data analysis

All data were analysed using the Statistical Package for Social Science (SPSS) software (version 22.0, IBM, New York, NY, USA).

The Mann-Whitney U test was used to compare ranked data between different groups. Student’s *t*-test was used for comparison of numeric data. The significance level was set at *p* < 0.05.

## Results

A total of 101 students in three classes were included in this study; 40 students were enrolled in the experiential education group and 61 in the control group. All three classes were comparable in terms of students’ age, gender and grade point average (*p*>0.05) (Table 1).

**Table 1:** The age, gender and GPA of the two groups

	Experiential education group	Control group	Significance level
Age	22.56 ± 0.68	22.42 ± 0.70	NS
Gender	24 M/17F	32 M/28F	NS
GPA^a^	3.11 ± 0.52	3.21 ± 0.36	NS

*NS* not statistically significant

^a^The GPA was calculated by the rule made by the Sun Yat-sen University. The data was acquired directly from the education department of Sun Yat-sen University. The full mark is 5

### Assessment result

The final examination was attended by a total of 99 students (the other 2 student delay the exam due to personal reasons), including 38 students in the experiential education group and 61 students in the control group. There were no significant differences in baseline grade averages between the two groups (Mann- Whitney U test, U = 1240.5, *p* = 0.614).

**Fig. 1:**
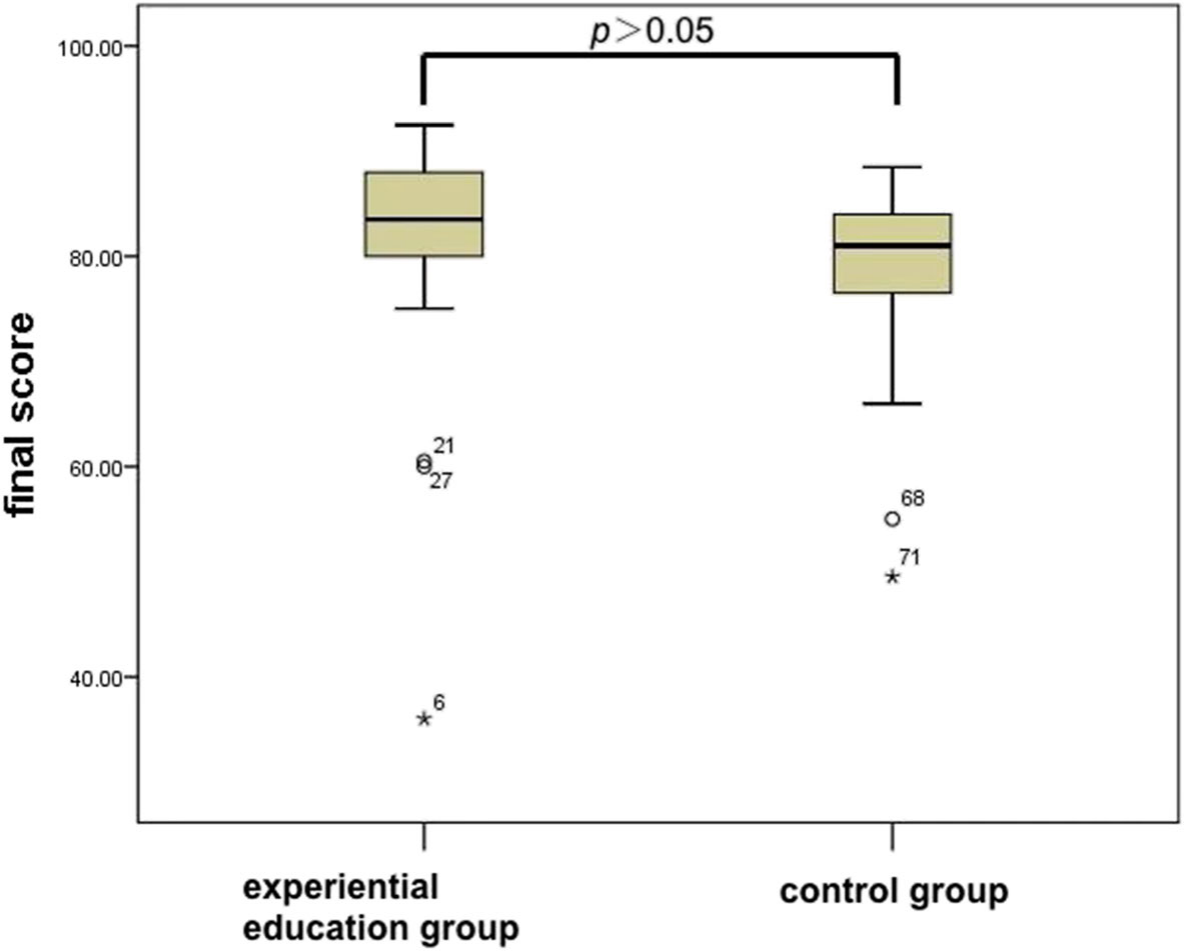
Comparison of total scores in final examination between the experiential education group and the control group

**Fig. 2:**
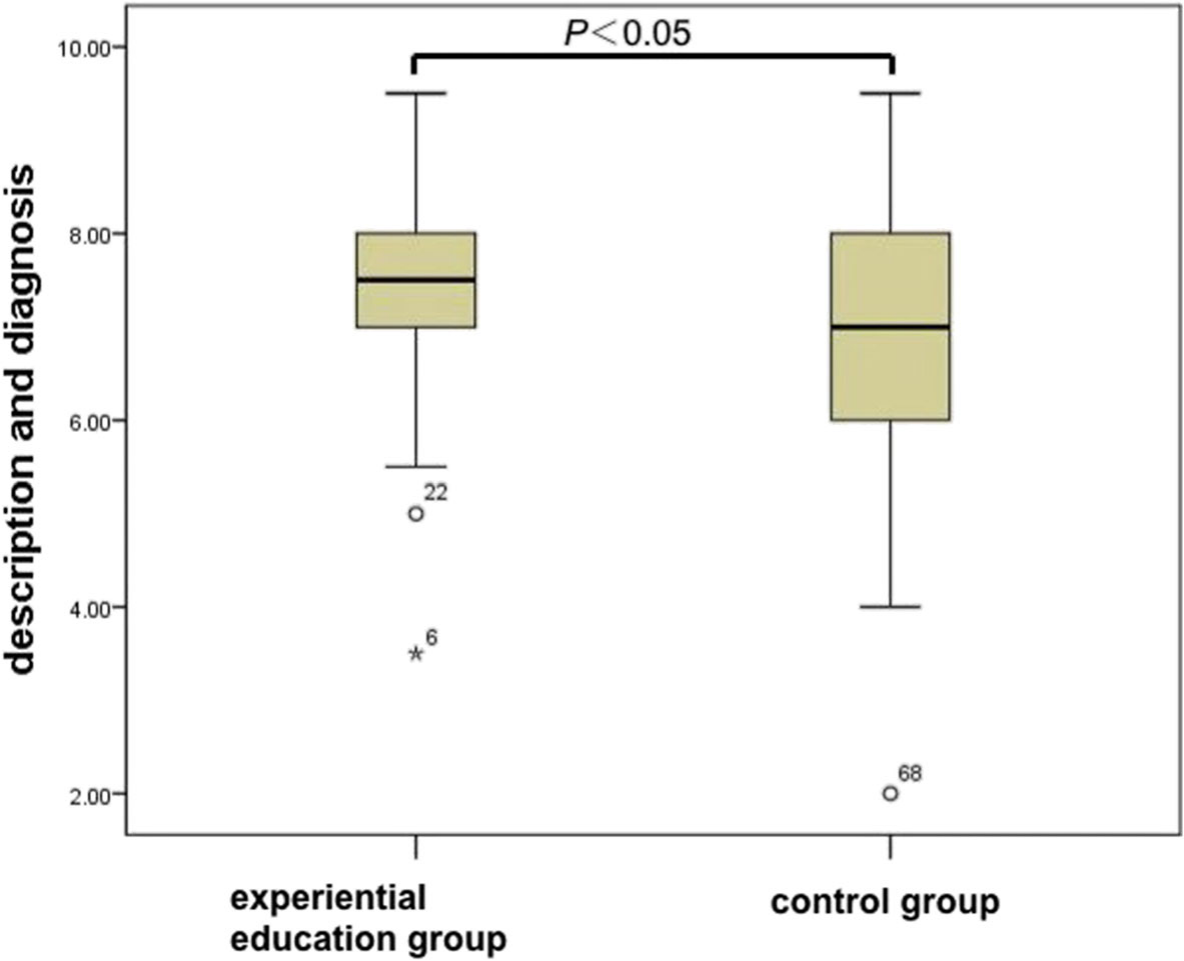
Comparison of scores in “Image Description and diagnosis” short-answer type questions between the experiential education group and the control group

The average score of the experiential education group was 81.5 ± 10.3, which was not significantly different from the control group (79.2 ± 7.5, *p* > 0.05) (Fig. 1). However, sub-domain analyses indicated a significant (*p* < 0.05) difference between the scores in “image description and diagnosis” short answer type questions. The experiential education group demonstrated significantly higher scores than the control group ((7.4 ± 1.3 and 6.7 ± 1.5, respectively, *p* < 0.05). In comparison, there were no significant differences in the scores for multi-choice questions (MCQs) between the two groups. (34.0 ± 4.8 in the experiential education group and 33.0 ± 3.4 in the control group, *p* > 0.05) (Fig. 2).

### Feedback results

Responses to the Likert scale statement are presented in Tables 2 and 3. All experiential education group participants and 47 control participants completed the self-assessment questionnaire. In comparison to the control group, the experiential education group had increased familiarity with each of the following: the DICOM viewer, the sequences and reconstruction methods of CT imaging, the diagnosis thinking principle, and anatomy. These findings serve to strengthen students’ self-confidence in their ability to perform future clinical work. (Table 2).

**Table 2:** Self-assessment Likert scale responses for the experiential education group and the control group

Likert scale questions	experiential education group*	control group*	Significance level (*p* value)
1. I am familiar with the basic functions and operations of the DICOM viewer software.	3.35 ± 0.10	2.30 ± 0.12	< 0.001
2. I am familiar with the basic CT scanning sequences.	3.60 ± 0.11	2.64 ± 0.14	< 0.001
3. I am familiar with the reading sequence of CT imaging.	3.35 ± 0.11	2.55 ± 0.14	< 0.001
4. I am familiar with the reconstruction methods of CT images.	3.48 ± 0.15	2.85 ± 0.15	< 0.001
5. I clearly understand how to adjust the proper window width and window level for observation.	3.50 ± 0.12	3.38 ± 0.11	0.004
6. I am familiar with the density of different tissue to choose an appropriate window width and window level.	3.55 ± 0.10	3.15 ± 0.11	NS
7. I am familiar with the location of different organs in the cross section.	3.38 ± 0.10	3.17 ± 0.12	0.016
8. I am familiar with the relative location of different organs, and I can reconstruct them in my mind.	3.20 ± 0.12	2.77 ± 0.13	NS
9. I have confidence in reading the CT images in the internship.	4.35 ± 0.10	3.89 ± 0.14	0.047
10. I agree that using the DICOM viewer can be helpful for learning clinical imaging.	4.18 ± 0.10	3.94 ± 0.13	0.024
11. I am interested in radiology.	3.23 ± 0.17	3.15 ± 0.11	NS
12. I think I may become a radiologist.	2.95 ± 0.13	2.85 ± 0.13	NS

*NS* not statistically significant

* The average score was calculated by the following valuation: strongly agree = 5; agree = 4; neutral = 3; disagree = 2; strongly disagree = 1

**Table 3:** The Likert scale questionnaire on learner satisfaction

1. The experiential education can increase my interest of radiology.					
	Strongly agree	Agree	Neutral	Disagree	Strongly disagree
Number of responders	11	23	4	2	0
Percentage of responders	27.5%	57.5%	10%	5%	0%
2. I am satisfied with the organization of the experiential education.					
	Strongly agree	Agree	Neutral	Disagree	Strongly disagree
Number of responders	11	22	7	0	0
Percentage of responders	27.5%	55%	17.5%	0%	0%
3. I am satisfied with the interactivity of the experiential education.					
	Strongly agree	Agree	Neutral	Disagree	Strongly disagree
Number of responders	9	25	5	1	0
Percentage of responders	22.5%	62.5%	12.5%	2.5%	0%
4. This kind of learning activity is easily accepted.					
	Strongly agree	Agree	Neutral	Disagree	Strongly disagree
Number of responders	9	23	8	0	0
Percentage of responders	22.5%	57.5%	20%	0%	0%
5. The experiential education can consolidate my knowledge of anatomy.					
	Strongly agree	Agree	Neutral	Disagree	Strongly disagree
Number of responders	14	22	3	1	0
Percentage of responders	35%	55%	7.5%	2.5%	0%
6. The knowledge is more easily accepted via experiential learning.					
	Strongly agree	Agree	Neutral	Disagree	Strongly disagree
Number of responders	13	21	6	0	0
Percentage of responders	32.5%	52.5%	15%	0%	0%
7. The experiential learning increased my understanding of the different imageological methods.					
	Strongly agree	Agree	Neutral	Disagree	Strongly disagree
Number of responders	11	22	6	1	0
Percentage of responders	27.5%	55%	15%	2.5%	0%
8. The experiential learning increased my confidence to face future clinical work.					
	Strongly agree	Agree	Neutral	Disagree	Strongly disagree
Number of responders	10	19	11	0	0
Percentage of responders	25%	47.5%	27.5%	0%	0%
9. The experiential education can increase my understanding of daily work in the radiology department.					
	Strongly agree	Agree	Neutral	Disagree	Strongly disagree
Number of responders	15	19	5	1	0
Percentage of responders	37.5%	47.5%	12.5%	2.5%	0%
10. Overall, I am satisfied with the quality of this learning activity.					
	Strongly agree	Agree	Neutral	Disagree	Strongly disagree
Number of responders	15	19	6	0	0
Percentage of responders	37.5%	47.5%	15%	0%	0%

All 40 students in the experiential education group provided feedback via additional self-assessment questionnaire specific to the experiential education. The analysis demonstrated that the majority of students were satisfied with the organization (82.5%) and interactivity (85%) of the learning activity. Most students found this kind of learning activity to be helpful for both learning radiology (85%) and understanding anatomy (90%). More importantly, a large proportion of students (85%) found that the experiential education encouraged better personal interest in radiology, as well as satisfaction with the quality of learning (85%). (Table 3).

Many students also reported benefitting from the experiential education via the free text responses on the questionnaire.

## Discussion

Traditional hands-on radiology education that continues to be used today only displays typical imaging layers rather than the whole images. While this teaching method may be useful for helping students handle typical imaging features, it may be insufficient for learning anatomy [14]. Hence, students may remain unable to provide quality image readings when they were expected to perform independently during clinical practice [15]. Although a variety of radiology education models such as problem-based learning [16] and the use of dynamic images can solve part of this problem, we believe the original working environment represents the most ideal learning method. Thus, we have introduced the experiential education method into our radiology teaching.

The theory of experiential education was first proposed by John Dewey in 1938. He initiated the topic of experiential education in his work entitled *Experience and Education*. Unlike hands-on education, this educational philosophy emphasizes the process of learning through experience [17]. Based on this educational concept, students should be responsible for their own learning. As such, students are able to acquire relative knowledge in the real world by discovering both questions and proactive solutions. This kind of learning method has the potential to motivate students’ autonomy while also elevating their interest of knowledge [17]. Outdoor education, cooperative and environmental learning each represents different practice models of experimental education. In a sense, the intern and resident rotation is also a kind of experiential education. This educational concept is increasing in popularity at all levels of education [18, 19].

During this study, we created an experiential education course by applying the PACS and DICOM viewer software to simulate a working environment mirroring our typical clinical work. The study results indicated the experiential education approach allows better clinical guidance necessary in assisting students to form a holistic point of view in both anatomy and pathology. Most importantly, this teaching method allows better guidance for students to develop critical thinking and systematic approach to formulate imaging interpretation and differential diagnosis, which may be partly thanks to the free inquisitive space of the experiential education mode.

Apart from objective improvement in imaging descriptions and interpretations, subjective improvements in self-confidence were also seen from the student feedback obtained during self-assessment questionnaires. Such skills included determining the order in which to read an imaging sequence, choosing the proper window width and level, as well as the choice of the reconstruction method. That might the result affected by the intervention of the trainer during the activity as well as the open discussion training. Moreover, following the experiential courses, the experiential approach allows better interactions which encouraged better interest in radiology which is vital for the future development of radiology [20].

Our study shows the efficacy of experiential education mode in the study of imaging anatomy. Anatomy is the basis for radiology education. In theory, reading CT and MR images is a good way to study anatomy because the contiguous scanning helps students to form three-dimensional concepts of relative locations of organs [21, 22]. It was globally concluded that imaging anatomy enhanced the quality and efficiency in human anatomy education [23]. However, it is hard to recognize the whole anatomical structure from a single cross-sectional image, which tends to increase student confusion [22]. Our study results provide evidence that reading a contiguous scan improves students’ comprehensive understanding of anatomy. Additionally, by utilizing multiple reconstruction methods, three dimensional images are more comprehensively visualized by students, which is a finding that has also been proven by other studies [24].

Much effort is needed to bring experiential education into practice. The PACS and a proper DICOM viewer represent the basic software requirements for experiential education. To protect patients’ privacy, we chose to copy the DICOM data from the PACS rather than to link to the original PACS. In this way, the development of a simulation PACS for undergraduate medical education similar to that of the University of Colorado School of Medicine is an ideal method for forming a simulation software environment [6]. In addition, teacher guidance is an especially critical element in education. At least 3 teaching assistants with standardized radiology training experience are needed in one class, as team-based discussion is a component in our experiential courses. Students need the teaching assistants to both guide image reading as well as to answer questions. Therefore, teaching assistants need specific experience working in a radiology department. Thus, we chose the junior radiology specialists as teaching assistant. Nevertheless, a shortage of teachers hinders the use of this teaching model on a wider scale, which serves as a limitation of the experiential education approach.

There are several limitations to the study. Firstly, due to the limited number of supervisors, the sample size was similarly limited. Secondly, this was a single centre study. Thirdly, due to the limitation of actual operation, only 47 of 61 students completed the questionnaire in the control group. Though the probability is very small, it still has a chance to lead to the bias of the result. Fourthly, though we have control the faculty and the teaching standard between the two groups, the bias caused by human factor still can not be fully avoided in practice. Fifthly, although we utilized objective evaluation measurements, this study also exposed the weakness of our evaluation system within radiology education. The study measures consisted of paper-and-pencil tests, with most questions consisting of objective items that test memory such as multiple choice questions and short answer questions. Furthermore, the subjective items that are used to test application ability are limited. Consequently, only a small part of the final exam reflected the difference between the experiential education group and the control group. Other test forms such as bedside examinations and multi-station examinations should be used in the future for better assessment of application ability [25, 26].

As stated in the students’ recommendations, this model of experimental teaching can still be improved. For example, at Dartmouth-Hitchcock Medical Center, students are required to attend a radiology triage programme to work with on-call radiology residents [27]. Such students have reported this to be a valuable clinical learning experience, as well as a good way to relieve the workflow of residents. In our questionnaire, some students also requested to take the internship in the radiology department. This kind of programme can be brought into practice as an important aspect of experiential education. Additional forms of education, such as integrative teaching, may also be applied in future radiology education courses [28].

## Conclusion

In conclusion, our study found that experiential education shows some advantages than traditional education model in improving analysis and diagnostic skills, as well as students’ self-confidence. As an attempt to bring the experiential education mode into undergraduate radiology education, we hope it will pave a new way for the education evolution.

## Supplementary information

**Additional file 1.** Self-assessment radiologic skills questionnaire (English language version).

**Additional file 2.** Learner satisfaction questionnaire (English language version).

## Data Availability

The datasets used and/or analysed during the current study are available from the corresponding author on reasonable request.

## Abbreviations

3D: 3-dimensional
CT: Computed tomography
DICOM: Digital Imaging and Communication in Medicine
MPR: Multiple planar reconstruction
MR: Magnetic resonance
PACS: Picture archiving and communication system
