# Supplementary material for: Constructing an experiential education model in undergraduate radiology education by the utilization of the picture archiving and communication system (PACS)
Source: BMC Med Educ. 2019 Oct 21;19:383. doi: 10.1186/s12909-019-1827-0 (PMC6805614; doi:10.1186/s12909-019-1827-0)
Supplement: Supplementary file 1 — Additional file 1. Self-assessment radiologic skills questionnaire (English language version). [file 12909_2019_1827_MOESM1_ESM.docx]

Self-assessment Radiologic Skills Questionnaire

**Please choose the one which is the most consistent with your situation.**

1. I am familiar with the basic functions and operations of the DICOM viewer software.

Strongly agree Agree Neutral Disagree Strongly disagree

2. I am familiar with the basic CT scanning sequences.

Strongly agree Agree Neutral Disagree Strongly disagree

3. I am familiar with the reading sequence of CT imaging.

Strongly agree Agree Neutral Disagree Strongly disagree

4. I am familiar with the reconstruction methods of CT images.

Strongly agree Agree Neutral Disagree Strongly disagree

5. I clearly understand how to adjust the proper window width and window level for observation.

Strongly agree Agree Neutral Disagree Strongly disagree

6. I am familiar with the density of different tissue to choose an appropriate window width and window level.

Strongly agree Agree Neutral Disagree Strongly disagree

7. I am familiar with the location of different organs in the cross section.

Strongly agree Agree Neutral Disagree Strongly disagree

8. I am familiar with the relative location of different organs, and I can reconstruct them in my mind.

Strongly agree Agree Neutral Disagree Strongly disagree

9. I have confidence in reading the CT images in the internship.

Strongly agree Agree Neutral Disagree Strongly disagree

10. I agree that using the DICOM viewer can be helpful for learning clinical imaging.

Strongly agree Agree Neutral Disagree Strongly disagree

11. I am interested in radiology.

Strongly agree Agree Neutral Disagree Strongly disagree

12. I think I may become a radiologist.

Strongly agree Agree Neutral Disagree Strongly disagree
