# Supplementary material for: Constructing an experiential education model in undergraduate radiology education by the utilization of the picture archiving and communication system (PACS)
Source: BMC Med Educ. 2019 Oct 21;19:383. doi: 10.1186/s12909-019-1827-0 (PMC6805614; doi:10.1186/s12909-019-1827-0)
Supplement: Supplementary file 2 — Additional file 2. Learner satisfaction questionnaire (English language version). [file 12909_2019_1827_MOESM2_ESM.docx]

Learner Satisfaction Questionnaire

**Please choose the one which is the most consistent with your situation.**

1. The experiential education can increase my interest of radiology.

Strongly agree Agree Neutral Disagree Strongly disagree

2. I am satisfied with the organization of the experiential education.

Strongly agree Agree Neutral Disagree Strongly disagree

3. I am satisfied with the interactivity of the experiential education.

Strongly agree Agree Neutral Disagree Strongly disagree

4. This kind of learning activity is easily accepted.

Strongly agree Agree Neutral Disagree Strongly disagree

5. The experiential education can consolidate my knowledge of anatomy.

Strongly agree Agree Neutral Disagree Strongly disagree

6. The knowledge is more easily accepted via experiential learning.

Strongly agree Agree Neutral Disagree Strongly disagree

7. The experiential learning increased my understanding of the different imageological methods.

Strongly agree Agree Neutral Disagree Strongly disagree

8. The experiential learning increased my confidence to face future clinical work.

Strongly agree Agree Neutral Disagree Strongly disagree

9. The experiential education can increase my understanding of daily work in the radiology department.

Strongly agree Agree Neutral Disagree Strongly disagree

10. Overall, I am satisfied with the quality of this learning activity.

Strongly agree Agree Neutral Disagree Strongly disagree
